# The influence of carbon emission trading on the optimization of regional energy structure^☆^

**DOI:** 10.1016/j.heliyon.2024.e31706

**Published:** 2024-05-23

**Authors:** Songbo Jia, Xiaowen Zhu, Xin Gao, Xiaotong Yang

**Affiliations:** a School of Finance, Henan University of Economics and Law, Zhengzhou, Henan, 450000, China; b School of International Trade and Economics, Anhui University of Finance and Economics, Bengbu, Anhui, 233017, China

**Keywords:** Carbon emission right trading, Energy structure, Green finance, Environmental regulation

## Abstract

The optimization of the energy structure is a crucial element in the development of green and low-carbon economies and societies. Market-based environmental regulations, such as carbon emissions trading, play a significant role in facilitating this transition. This study analyzes panel data from 30 provinces in China from 2007 to 2020. It empirically examines the impact and mechanism of carbon emissions trading pilot schemes on the transformation of energy consumption structure at the macro level, using the difference-in-differences method. The research findings indicate that provinces with carbon emissions trading pilots have experienced significant reductions in carbon emissions and carbon intensity, demonstrating clear emission reduction effects. Additionally, the transformation process of energy consumption structure has been notably accelerated, highlighting the correlation between initial carbon emissions trading and the regional energy utilization framework. Furthermore, in our extended mechanism analysis, we introduce two new variables - green finance and environmental regulation. Our analysis reveals that the level of green finance in carbon emissions trading pilot provinces significantly influences the degree of carbon emissions in these areas. Additionally, environmental regulation has a significant positive impact on the optimization of energy consumption structure.

## 1 Introduction

The issue of climate change, caused primarily by greenhouse gas emissions such as carbon dioxide, has garnered considerable attention globally [1]. Studies from international climate science agencies reveal that the Earth's average atmospheric temperature has increased by approximately 0.85 °C over the past century since the onset of industrialization. This rise in temperature is attributed to the growing concentrations of greenhouse gases in the atmosphere resulting from human activities, and it poses significant challenges, including global warming [2]. The Chinese government has actively responded to climate change by improving the carbon emission trading market system, emphasizing that green development equals economic development, and improving the environment means developing productivity. China implemented a carbon trading pilot in 2011 and launched the first carbon emissions trading platform in Shenzhen in 2013, marking a crucial step in the construction of China's carbon trading market. Since then, carbon trading pilots have been conducted in various provinces and cities in China, and after over a year of development, the rules of the carbon trading market have gradually improved.

China's energy consumption is shaped by its abundant coal resources and limited oil and gas reserves. Coal accounts for a significant proportion of its energy consumption, while oil, gas, and non-fossil fuels have smaller shares [3]. In 2022, China's total energy consumption reached 5.41 billion tons of standard coal, with coal representing 56.2 % and clean energy comprising 25.9 %. Fossil fuels, particularly coal, remain dominant in the energy mix. However, coal combustion leads to high carbon emissions and the generation of pollutants like particulate matter, sulfur dioxide, and nitrogen oxides, contributing to atmospheric pollution. Consequently, transitioning China's energy consumption structure from fossil fuels to clean and low-carbon sources is urgently needed [4]. Prioritizing the development of clean energy, accelerating the shift from traditional to clean energy sources, and promoting the integration of energy with other sectors are essential for achieving sustainable development in the energy industry. Carbon emissions trading, as a market-based environmental management tool, plays a crucial role in achieving national carbon peaking and carbon neutrality goals and has a significant impact on optimizing regional energy structures. Currently, the carbon emission trading practice in China is at a crucial juncture. It is imperative to investigate whether the operation of China's carbon trading pilot scheme is beneficial for enhancing the optimization of the regional energy structure. Furthermore, additional research is required to explore the action pathway and the resulting impact on the optimization of the regional energy structure.

## 2 Literature review

The study of carbon emissions, a prominent aspect of emissions trading, has received considerable attention since the introduction of Coase Theorem. This theorem, which focuses on market mechanisms to address externalities in environmental pollution, has paved the way for extensive research in this field [5]. That is, under the premise of clear property rights, resources can achieve optimal allocation through market transactions. Currently, a large number of studies focus on two aspects: the construction and institutional design of carbon market systems, as well as the evaluation of policy effectiveness. One category of literature qualitatively explores the necessity of developing a carbon market in China and studies the effectiveness of policy practices by referencing the experiences of developed countries. This scholarly analysis involves a comparative examination of China's carbon trading market in relation to foreign markets, aiming to uncover the challenges and growth prospects confronted by China's carbon market development [6]. Additionally, it delves into the synergistic mechanism between carbon tax and carbon trading policies, adopting a multi-source theory perspective [7]. Another area of literature explored in this context pertains to the investigation of the mechanism behind the emission reduction effects and economic impacts of carbon trading.

In terms of environmental impact, the attention of international scholars studying carbon emissions trading systems has primarily been directed towards the development of pertinent mechanisms. It is widely acknowledged that the concept of carbon emissions trading emerged as an evolution of emissions trading rooted in pollution rights. By establishing clear and defined emission rights for companies, the objective is to internalize the external costs stemming from corporate carbon emissions. Through spontaneous trading of emissions rights among companies, an optimal allocation of emissions among companies can be achieved, ultimately leading to an overall carbon reduction effect. Unlike traditional command-and-control environmental policies, the government does not directly interfere with corporate behavior but guides companies to make decisions through market signals to achieve emission reduction targets [8]. [9] also theoretically proved that among various emission reduction methods, market-based emissions trading has the lowest emission reduction costs in a perfectly competitive market. With the advancement and refinement of China's carbon market trading policies, there has been a growing body of empirical research on China's carbon market. Numerous studies have verified that carbon trading policies have a significant impact on curbing carbon emissions from industrial production [10]. Moreover, these policies have been found to decrease energy consumption and carbon emission intensity in regulated industries within pilot regions [11]. Additionally, carbon trading policies have demonstrated cross-border emission reduction and neighboring demonstration effects [12]. From an economic standpoint, carbon emissions trading can foster integrated economic and environmental development, thereby promoting the adoption of green and low-carbon practices within regional economies [13,14]. This is primarily achieved through incentives for low-carbon technological innovation [15] and the promotion of industrial structure upgrading [16]. Furthermore, a notable advantage of carbon emissions trading instruments is their ability to effectively incentivize the optimization of overall economic costs and benefits [17], while also reducing long-term emission reduction costs through the induction of climate-friendly technological changes [18].

In the realm of energy structure, the current body of literature primarily focuses on identifying the factors and mechanisms that impact energy structure in order to achieve optimization and formulate scientifically sound energy development plans. In a study conducted by Ref. [19], path analysis was employed to clarify the influencing mechanisms of factors such as energy consumption constraints, carbon emission constraints, economic growth, population, and industrial structure on energy consumption structure. The authors argued that energy consumption constraints and GDP growth are the primary factors that inhibit coal consumption growth and promote the optimization of primary energy consumption structure [20]. Another study used path analysis to examine the influencing factors of energy consumption structure. The researchers found that carbon emission constraints directly impact the optimization of energy consumption structure, while energy consumption constraints, economic development level, energy prices, and energy endowments indirectly contribute to the optimization of energy consumption structure. Scholars both domestically and internationally have investigated the influencing factors of energy structure using various technical methods, and it can be concluded that the main influencing factors of China's energy structure include economic growth, energy consumption constraints, carbon emission constraints, industrial structure, energy prices, and population [21]. Furthermore, studying the characteristics and causes of China's energy structure and analyzing the existing issues within it hold significant theoretical and practical implications for the transformation of China's energy structure. According to Ref. [22], the problems within China's energy structure can be attributed to disparities in energy endowments, societal development needs, and the neglect of environmental and social costs across different regions [23]. posited that the imbalance in energy structure stems from an imbalance between supply and demand, and suggests that reducing the proportion of coal usage, increasing the proportion of oil and gas, and adjusting the supply structure are necessary steps to achieve a balance between energy supply and demand.

A number of studies have been conducted on the topic of this article, which examine the correlation between carbon emission trading pilot policies and various aspects of energy consumption, such as total energy consumption [24], energy consumption intensity [25], energy efficiency [26], energy investment [27], and the development of renewable energy [28]. These studies have found that carbon emission trading pilot policies have the potential to significantly reduce both the overall amount and intensity of energy consumption in a given region. Additionally, these policies can enhance the efficiency of energy use across different factors and promote investment in low-emission power generation technology projects in pilot areas. Furthermore, they generally contribute to the overall development of renewable energy sources. Currently, there is substantial research attention on the effects of carbon emission trading policies on emission reduction and economic factors, as well as the optimization and transformation of energy structures. However, there is still a lack of literature that specifically examines the impact of carbon emission trading policies on the transformation of regional energy structures. This research gap is attributed to two main deficiencies. First, the existing policy research mainly focuses on micro-level analysis, local areas, or single-factor productivity, lacking a unified theoretical framework that explains the mechanism of carbon trading policies on micro-level actors. Second, the mechanism research on the optimization of regional energy structures by carbon trading policies lacks in-depth exploration. The current literature attributes the effect of carbon trading policies on regional energy structures to either a single mechanism or the parallel influence of multiple mechanisms, neglecting the interplay between these mechanisms.

The contributions of this paper can be identified in several aspects：Firstly, it examines the influence of carbon emissions trading pilots on the optimization of regional energy structures from a macro perspective. The analysis combines regression analysis and testing with indices related to oil and gas substitution for coal and non-fossil energy substitution. This approach broadens the research scope of existing literature on carbon emissions trading policies and enhances the understanding of the implementation effects of such policies. Secondly, the optimization of energy consumption structure is crucial for China's economy to achieve green development. This paper partially addresses the research gap in promoting the green and low-carbon transformation of the economy and society. It provides theoretical support and empirical evidence for the timely achievement of the "double carbon" target and the promotion of green economic development. Thirdly, the paper introduces various indicators to construct green finance indicators and utilizes entropy methods to measure green finance. Environmental regulation indicators are also employed to measure environmental regulation. This expands the measurement dimensions of green finance and environmental regulation. Lastly, the paper conducts an in-depth analysis of the regulatory mechanism of carbon emissions trading policies on the optimization of regional energy structures from the perspectives of environmental regulation and green finance. This expands the understanding of the internal mechanism of the impact of carbon trading pilot policies on the optimization of regional energy structures and provides practical evidence for further promoting the transformation of regional energy structure optimization.

## 3 Institutional background and typical facts

### 3.1 Institutional background

The concept of carbon emission trading, which can be traced back to the proposal of pollution rights trading by economists during the 1990s, has evolved into a significant market-oriented strategy for mitigating carbon emissions [29]. defined carbon emission trading as treating carbon dioxide emission rights as commodities, allocating quotas under a predetermined total carbon emission target. The utilization of this system enables corporations to engage in the procurement or disposition of carbon emission regulations within the market, contingent upon the expenses associated with the mitigation of emissions. In doing so, this system effectively promotes adherence to carbon reduction standards. The implementation of carbon emission trading has proven to be a successful strategy in merging economic and environmental progress, thereby promoting the growth of green and low-carbon economies within regional contexts [13,30]. This approach contributes to achieving energy efficiency and emission decrease in China at a lower social cost.

China's carbon emission trading trial initiative has undergone three distinct stages. The first stage involved completing the preparatory phase of the pilot policy. In 2011, the State Council and the National Development and Reform Commission (NDRC) granted approval for launching carbon emission trading pilots in seven cities, including Shenzhen, Shanghai, Beijing, Guangdong, Tianjin, Hubei, and Chongqing. This decision laid the groundwork for establishing a market for carbon emission trading. In 2012, the NDRC introduced the "Interim Measures for the Administration of Voluntary Greenhouse Gas Emission Trading," which outlined a mechanism for verifying voluntary emission reductions and specified the fundamental principles and procedures for carbon emission trading. The second phase marked the establishment of carbon emission trading markets in the pilot regions. In 2013, the carbon emission trading markets in these regions were officially launched to implement the pilot policy. In 2014, the NDRC released the "Interim Measures for the Administration of Carbon Emission Trading," presenting an initial framework for operating China's carbon emission trading market. Subsequently, in August 2016, the Central Committee of the Communist Party of China and the State Council issued the "Implementation Plan for the National Ecological Civilization Pilot Zone (Fujian)" to explicitly support the expansion of the carbon emission trading market pilot in Fujian Province. This pilot officially commenced operations in December of the same year. The third phase witnessed the nationwide launch of the carbon emission trading market, with online trading initiated. The power generation sector became the initial industry to participate in the national carbon market, making it the world's largest market for trading greenhouse gases in terms of carbon emissions. The transition from pilot operations to national construction of the carbon market is expected to accelerate the achievement of dual-carbon goals, which holds significant implications for the development of a new pattern of dual circulation.

The implementation of a consolidated nationwide carbon emissions trading market represents a pivotal measure in the regulation and mitigation of greenhouse gas emissions through market-based mechanisms, thereby facilitating the transition towards a sustainable and environmentally-friendly economy. It serves as a vital policy tool to strengthen the construction of ecological civilizations and to meet international commitments to reduce emissions. Carbon emission trading serves as an effective mechanism for promoting emission reduction, as indicated by research [31]. Furthermore, it has been found to enhance green total factor productivity through various means, such as improving investment efficiency [32] and fostering a greater market orientation among enterprises [33]. This approach particularly contributes to the improvement of green production performance for high-energy-consuming enterprises [34].

### 3.2 Typical facts

China's pilot programs in carbon emission trading are distinguished by their unique characteristics:

First, it is crucial to acknowledge that China's carbon market pilots are characterized by a rather restricted trading scope, resulting in a substantially lower volume of domestic carbon emission trading when compared to the European Union's carbon emission trading market. Specifically, in the year 2020, the cumulative transaction volume across the eight carbon emission trading pilots in China amounted to 58.85 million tons, translating to a daily average transaction volume of 356,700 tons. In stark contrast, the European Union's carbon emission trading market experienced a substantially higher daily average transaction volume, reaching a staggering 3 million tons during the same period.

Second, in comparison to the European Union, the cost of trading carbon emissions in China is significantly lower on average. By 2022, the average transaction price in eight carbon emission trading pilots in China was 45.61 yuan per ton, while the lowest spot settlement price for European Union carbon emission quotas in 2022 was 57.92 euros per ton, reaching nearly 100 euros per ton at its highest.

Third, there are significant variations in transaction volume and average transaction price between different carbon emission trading pilots. In terms of transaction volume, Guangdong Province, which initiated its pilot program in December 2013, boasts the largest scale among all national carbon market pilots. In 2022, Guangdong Province led both in transaction volume, 14.61 million tons, and in transaction amount, 1.03 billion yuan. Regarding the average transaction price, Beijing Municipality has consistently held the top position in transaction prices among pilot regions since 2017. In 2022, Beijing achieved the highest average transaction price at 149.00 yuan per ton, with a range from 41.51 yuan per ton to 149.00 yuan per ton. In contrast, Shenzhen's average transaction price ranged from 4.08 yuan per ton to 65.98 yuan per ton during the same period. The variation in the amount and mean cost of transactions across various carbon emission trading initiatives underscores the diverse impacts of these pilot policies in different areas, presenting crucial practical consequences for the seamless achievement of dual-carbon objectives.

The distribution of energy structures in Chinese regions is characterized by the following characteristics.

The main source of energy in China's primary energy system is primarily derived from coal. Despite increases in the utilization of hydropower and other renewable energy sources, coal consumption still constitutes an excessively high proportion of the energy mix. In 2007, coal consumption represented a staggering 69.46 % of China's primary energy structure, with petroleum contributing 20.12 %, natural gas 3.34 %, and nuclear power a mere 0.76 %. Although India's energy structure is also coal-centric, its coal consumption proportion is lower at 51.43 % compared to China. In particular, the proportions in the United States and Japan are both below 25 %. China's rapid economic development demands a substantial supply of inexpensive energy, and the resource and price advantages of coal make it the preferred choice, thus forming the primary characteristic of China's energy structure. Although coal is relatively inexpensive, it emits the highest amount of carbon dioxide. In the year 2007, China made a significant contribution to atmospheric carbon dioxide emissions by releasing a staggering 6.72 billion tons, accounting for 24.3 % of the global total. This surpassed the United States, propelling China to the top position as the foremost contributor to the accumulation of carbon dioxide in the Earth's atmosphere.

In terms of analysis of energy consumption intensity, within the sample period, China displays a clear pattern of being "elevated in the north, reduced in the south," and "elevated in the west, diminished in the east." The observed trend aligns with the differing degrees of economic progress across various areas. Areas with higher economic development tend to have a more rational industrial structure and higher energy efficiency, resulting in a lower intensity of energy usage.

Reflecting on the pattern of energy usage from 2007 onwards, China has consistently exhibited a pattern of "increased energy in the north, decreased in the south," and "increased energy in the central area, reduced in the east and west." The observed trend is intimately linked to the coal deposits found across different provinces. The majority of China's coal reserves are clustered in areas including Shanxi, Shaanxi, Inner Mongolia, and Henan. The statement implies, albeit indirectly, that the distribution of resources, including coal, significantly influences the energy consumption patterns within a particular region.

Based on the delineated features of carbon emissions and regional energy structures, this scholarly investigation undertakes an examination of the impacts that carbon emissions trading has on the aforementioned regional energy structures. The objective is to expand the scope of research regarding the success of pilot programs in carbon emission trading.

## 4 Theoretical foundation and mechanism hypotheses

As a carbon pricing tool, the carbon emission trading policy, compared to command-and-control regulations, has strong flexibility and long-term incentives, promoting energy efficiency and energy structure improvement [35].Initially, the carbon trading market will drive the rapid advancement of clean energy. Through the carbon trading policy, carbon emission rights are treated as commodities, subject to the limitations of carbon emission quotas and the objective of pursuing maximum profitability. Consequently, enterprises will need to progressively reduce their reliance on high-carbon energy sources, such as coal, while increasing the utilization of cleaner alternatives like natural gas, hydropower, and nuclear power. Moreover, there will be a greater emphasis on investing in renewable energy projects to enhance economic output per unit of energy consumption. This momentum will encourage further investment in renewable energy initiatives and contribute to improved economic output in relation to energy consumption. The substitution effect of clean energy is increasingly evident within the power industry, and the introduction of the carbon trading market offers multiple advantages for the development of clean energy. Secondly, the carbon trading policy will expedite the process of phasing out coal-fired power generation capacity. In response to the formidable challenges posed by climate change, the global trend of "abandoning coal" has emerged. Presently, over 30 countries, including Germany, the United Kingdom, and the Netherlands, have established timetables for discontinuing coal usage. Additionally, major carbon-emitting nations, such as the European Union, China, Japan, and South Korea, have committed to achieving carbon neutrality by roughly the middle of this century. Since 2020, China has consecutively exceeded 100 million kilowatts of newly installed wind and solar power capacity, accounting for approximately 60 % of the total new installed generation capacity, gradually replacing coal-fired power generation. Finally, the carbon emission trading market will facilitate the transformation of coal-fired power companies. Currently, the power generation industry dominates the carbon trading market, with a particular focus on coal-fired power companies that emit significant amounts of carbon. The combustion of coal generates substantial greenhouse gases, leading to severe environmental pollution. While power generation already necessitates meeting certain standards, the promotion of the carbon trading policy will compel coal companies to improve their efficiency in utilizing carbon-based energy, alter their power structure, and transition towards clean energy generation, such as photovoltaic or wind power. This transformation will result in an increased proportion of clean energy generation and the transition of these companies into clean energy power entities.

In contemporary times, researchers have redirected their attention from the national domain to the local domain while investigating the correlation between China's low-carbon economy and the regional energy economic structure. This shift involves merging comprehensive analyses with the economic and industrial conditions of specific regions. In their scholarly investigation [36], undertook a comprehensive examination of the present condition of low-carbon economic advancement in Yantai, Shandong. This study involved a comparative analysis, juxtaposing the low-carbon city initiatives in Yantai with those implemented in renowned urban centers like London and Tokyo. By considering the resource advantages and geographical locations of each city, he explained how carbon reduction measures can drive industrial structure changes in Yantai and promote the mechanism of sustainable local economic development [37]. selected ten provinces with the highest number of China's CDM carbon transactions as sample regions. Using panel data models based on indicators such as the estimated carbon emission reductions from CDM projects announced from 2006 to 2012 and the proportion of regional industrial structure, she empirically analyzed that the size of carbon trading volume will significantly affect industrial structure changes. Her conclusion was that CDM carbon trading can positively impact the changes in the regional industrial structure coefficient. Hence, propelled and bolstered by carbon trading measures, the establishment of a varied and robust low-carbon energy supply system, alongside an effective and environmentally-friendly energy consumption system, can facilitate the enhancement and advancement of China's regional energy framework. In light of this, the present scholarly article puts forth the suggestion.

Hypothesis 1 The carbon emission trading pilot policy is conducive to the optimization of regional energy structure.

In 2016, the issuance of the "Guiding Opinions on Building a Green Financial System" by the People's Bank of China served to provide a clear definition of green finance. It encompasses financial services that are directed towards project financing, project operations, risk management, and other relevant aspects in the fields of environmental protection, energy conservation, clean energy, green transportation, and green buildings. This definition encompasses various elements such as green credit, green securities, green insurance, and green investment. Green finance, as an innovative financial model, aims to direct capital flows towards low-carbon and environmentally sustainable development areas. This is achieved through the introduction of innovative financial products and services, as well as the establishment of relevant institutional arrangements. The ultimate goal is to achieve coordinated development between the environment, resources, and the economy, while also considering the economic benefits and ecological environment protection [38]. Furthermore, the promotion of green finance also plays a crucial role in optimizing and transforming energy structures. Firstly, green credit provides financial support for the optimization of energy structures. Green credit incentivizes commercial banks to issue green loans and increases the financing costs of "two remaining and one high" industries, effectively curbing the scale expansion of high-energy-consuming industries. It guides social capital into green industries, driving the development of low-carbon and energy-saving industries. For example, in 2018, Shanghai Pudong Bank initiated a special channel for financing green products for enterprises, addressing the difficulty of financing for enterprises producing low-carbon products. Secondly, green securities can improve the level of capital investment for energy structure optimization. Issuing green stocks and green bonds are more efficient ways to obtain financing, enabling enterprises to obtain more transformation funding support, helping to expand enterprise financing scales, optimize financing structures, and promote the rationalization and decarbonization of energy regions in high-energy-consuming industries. For example, after an enterprise issues green stocks, the rise in stock prices means that the enterprise will have more capital to raise in the market, making the reset cost of environmentally friendly energy enterprises lower compared to market prices, providing conditions for abandoning old equipment and purchasing new ones in production and operations and expanding investment scales. Green securities help energy industries expand enterprise financing scales, optimize financing structures, and promote the rationalization and decarbonization of energy regions in high-energy-consuming industries during the process of structural optimization. Thirdly, green insurance provides risk protection for the optimization of energy structures. Green insurance considers climate and the environment as more prominent risk factors, designing insurance guarantee mechanisms suitable for the energy industry, compensating for the deficiencies of traditional insurance in resisting risks during the optimization and transformation of energy structures [39]. Lastly, unlike gray investments, green investments, as investments in environmental protection, combine the rational use of resources, measures to prevent pollution, and production investments, effectively reducing resource consumption and environmental pollution, which is beneficial to sustainable socio-economic development.

In the realm of empirical research, local scholars have conducted a comprehensive analysis on the influence of green finance on energy structure. This analysis is grounded on the theoretical foundations of the dual carbon target and national finance, and employs data from 30 provincial regions in China. By utilizing the entropy method, the scholars were able to estimate both the green financial development index and the energy structure optimization index. The findings of this study indicate that China's green financial development indicators have displayed a fluctuating growth pattern, while the energy structure has been gradually optimized over time. Furthermore, it was observed that green financial development exerts a positive impact on energy structure optimization, which is mediated by financing scale and moderated by technological progress. Building upon these results, the present article proposes relevant implications and recommendations.

Hypothesis 2 The carbon emission trading pilot policy promotes the optimization of regional energy structure through the innovative effect of green finance.

Environmental regulation plays a vital role in achieving a balance between economic development and ecological harmony on a macro level. It has the power to influence the allocation of market resources and facilitate the optimization of energy structures by adjusting energy production methods, enhancing energy utilization, and shaping energy market operations. Firstly, there exists an "inverted U″ relationship between environmental regulation and energy efficiency [40]. To some extent, environmental regulation can have an inverse effect on local energy technology, compelling energy producers in the area to adopt cleaner and more sustainable production methods, thereby improving the energy structure within the region. Secondly, environmental regulation promotes the upgrading of energy consumption patterns. Through regulations, incentives, or penalties, environmental regulation encourages residents, enterprises, and public institutions to choose clean energy sources and energy-saving products, thereby enhancing the regional energy consumption structure. Thirdly, environmental regulation influences the functioning and price formation of energy markets. Carbon emission trading, as a market-based environmental regulation policy, guides the market towards cleaner and more sustainable energy choices, thereby optimizing the energy structure. Lastly, environmental regulation supports the development of renewable energy projects and energy transformation initiatives by providing financial support, tax incentives, and other measures, promoting the establishment of a more sustainable regional energy structure.

Carbon emission trading, as a market-based environmental regulation approach, aligns with the Porter Hypothesis principle, which asserts that appropriate environmental regulations can prompt enterprises to adopt and develop environmentally friendly technologies and products. This, in turn, reduces carbon emissions, avoids external costs resulting from environmental penalties, boosts the market competitiveness of enterprises, and facilitates the optimization and upgrading of the overall regional energy structure. A study conducted in the United States in 1977, which examined data from the 1960s–1970s, found that environmental regulation can lead to improvements in energy efficiency, with the economic benefits far outweighing the costs incurred [41]. Similarly, an analysis of the correlation between environmental regulation and energy consumption structure in China from 1998 to 2006 concluded that there is a significant negative correlation between the level of environmental regulation and the scale of coal consumption [42]. Building upon these results, the present article proposes relevant implications and recommendations.

Hypothesis 3 The pilot policy for carbon emission trading rights promotes the optimization of regional energy structures through the driving effect of environmental regulation.

## 5 Empirical research design

### 5.1 Empirical model

In October 2011, the relevant departments in China issued a notice on conducting pilot carbon emissions trading, marking a crucial step in the development of China's carbon trading market. Subsequently, starting from 2013, China has successively conducted pilot carbon emissions trading in seven provinces and cities including Beijing, Tianjin, Shanghai, Chongqing, Guangdong, Hubei, and Shenzhen. This study utilizes panel data from 30 provinces and cities in China from 2007 to 2020 with a focus on data availability. The experimental group consists of the aforementioned pilot provinces and cities while the control group comprises other provinces. The pilot phase spans from 2013 to 2020 representing the formal implementation of policies while the pre-pilot phase includes the years from 2007 to 2012.

In 2005, Chinese scholars Zhou Lian and Chen Ye employed the Difference-in-Differences (DID) model to conduct a systematic evaluation of the effectiveness of China's rural tax and fee reform policy, utilizing data from 591 counties in seven provinces [43]. This marked the first instance of domestic scholars employing the DID model to assess policy effectiveness. Building on the research methods employed in a previous study, the present study utilizes the DID model to investigate the impact of carbon emission trading policies on China's energy efficiency. The establishment of pilot areas, along with their variation in temporal implementation, serves as a crucial exogenous source of variation for examining the influence of carbon emission trading policies on energy structures. By employing the carbon pilot provinces (cities) as the basis for quasi-natural experiments, this study analyzes the effects of carbon emission trading policies on China's energy efficiency. The model can be expressed by Formula (1).

(1) $$Y_{i,t}=\beta_{0}+\beta_{1}{treat}_{i}\times {post}_{t}+\beta_{2}Control+\mu_{i}+e_{t}+\varepsilon_{i,t}$$

In the given model (1), where i represents the province and t signifies the year, the dependent variable is denoted as Y, which specifically refers to the energy structure coefficient. The term 'treat' denotes the variable for provincial grouping, 'post' indicates the variable for time grouping, and 'Control' refers to a collection of provincial control variables linked to the dependent variable. $\mu_{i}$ symbolizes the fixed-time regional effects, $e_{t}$ signifies the constant-time effects, and $\varepsilon_{i,t}$ denotes the variable error component.

### 5.2 Data source and processing

The current research utilizes macro panel data from 30 provinces in China, covering the period from 2007 to 2020, as the foundation for its analysis. Nevertheless, due to substantial data constraints in Tibet, as well as the Hong Kong, Macao, and Taiwan regions, these areas were not included in the study. In order to conduct mechanism testing, the pertinent firm-level data is obtained from a designated company database, while most of the other data is sourced from the CSMAR database, WIND database, EPS platform, and publications such as the "China Energy Statistical Yearbook," "China Environmental Statistical Yearbook," and various provincial statistical yearbooks. The authors manually compile a list of provinces involved in the pilot carbon emissions trading program from government-sanctioned websites. Detailed statistical information for each variable, including sample size, mean, standard deviation, as well as the minimum, median, and maximum observed values, are presented in Table 2.

#### 5.2.1 The dependent variable

The transformation of the energy consumption structure is an ongoing and complex endeavor, involving the continuous optimization and adjustment of various dominant energy sources through substitution and complementation. It is important to note that the carbon emission coefficients and production capacities of different energy sources vary significantly, and any changes in the energy consumption structure are closely tied to alterations in carbon emissions.

The promotion of the dual replacement process, specifically "oil and gas replacing coal, non-fossil energy replacing fossil energy," is not only necessary to transition from a high-pollution and carbon-intensive energy consumption structure to a clean and low-carbon one, but also crucial in the comprehensive construction of a modern energy system. To assess the degree of optimization in the energy structure, this paper introduces three energy structure replacement indices [44].

These indices include the Oil and Gas Replacement Index (OGE), the Non-fossil Energy Replacement Index (REE), and the Energy Structure Dual Replacement Index (TRE) which is synthesized from the former two indices. These indices represent the proportions of energy consumption from coal, oil, natural gas, and non-fossil sources in the overall energy usage. The following section provides a detailed explanation of the methodologies employed to develop these indices.

The Oil and Gas Substitution Index (OGE) is expressed as the ratio of the sum of the proportions of oil and natural gas consumption to the proportion of coal consumption, reflecting the degree of substitution of oil and gas for coal. Specifically, it can be expressed as:

(2) $$OGE=\left(E_{0}+E_{g}\right)/E_{c}$$

The Non-fossil Energy Replacement Index (REE) is calculated by dividing the amount of non-fossil energy consumed by the amount of fossil energy consumed. This ratio indicates the replacement of fossil energy with non-fossil energy and can be expressed as:

(3) $$REE=\frac{E_{n}}{E_{c}+E_{0}+E_{g}}=\frac{E_{n}}{1-E_{n}}$$

The derivation of the Dual Replacement Index of Energy Structure (TRE) involves the computation of the geometric mean of two distinct indices, namely the Oil and Gas Replacement of Coal Index (OGE) and the Non-fossil Energy Replacement Index (REE). Mathematically, it can be expressed as follows:

(4) $$TRE=\sqrt{OGE\times REE}=\sqrt{\frac{\left(E_{0}+E_{g}\right)\times E_{n}}{E_{c}\times \left(1-E_{n}\right)}}$$

This article refers to Ref. [44]. The explained variables of the paper are constructed, and three energy structure alternative indices are used to measure the degree of energy structure optimization. Formulas（2）-（4） correspond to the three indices constructed respectively. In formulas （2）-（4）, $E_{c}$, $E_{0}$, $E_{0}$ and $E_{n}$ respectively denote the proportions of coal consumption, oil consumption, natural gas consumption, and non-fossil energy consumption in the total energy consumption. These variables are subjected to a logarithmic transformation when analyzing the dependent variable.

#### 5.2.2 The explanatory variables

Carbon Emission Trading Pilot: In this scholarly study, the carbon emission trading pilot serves as a proxy variable for the carbon neutrality policy. The year 2011 witnessed the release of a notification by China's National Development and Reform Commission, outlining the initiation of pilot projects concerning carbon emission trading. However, the publication of emission control standards and the list of companies subjected to emission control in various pilot regions were primarily concentrated between 2012 and 2013. The inception of the carbon emission trading markets primarily occurred between 2013 and 2014. This study designates 2013 as the year that various pilot regions experienced policy impacts. Take into account the establishment of pilot provinces for carbon emission trading in China, including Beijing, Shanghai, Tianjin, Chongqing, Hubei, and Guangdong, as virtual variables, the period before 2013 is labeled as "pre-policy" (post0), and the period after 2013 is labeled "post-policy" (post1) [45]. Cities located in the pilot provinces receive "treatment" (treat1), while those in the nonpilot provinces are categorized as "untreated" (treat0).

Carbon Trading Price: Given that pilot cities that conduct "carbon trading" already have a carbon trading market with existing trading prices, this study further retrieves annual carbon trading prices from the Wind database. The annual average highest and lowest carbon trading prices are then calculated as additional indicators of the carbon neutrality policy for further stability testing.

#### 5.2.3 Moderating variable

To validate the Second and third hypotheses in the mechanistic analysis, this study intends to introduce two variables, namely, Green Finance (GFI) and Environmental Regulation (ER), as moderating variables for further analysis.

Green Finance (GFI): Green finance refers to the financial services provided by the banking industry, encompassing investment and financing activities, project operations, and risk management, to support environmental improvement, address climate change, and promote efficient utilization of resources. These services are specifically targeted towards projects in sectors such as environmental protection, energy conservation, clean energy, green transportation, and sustainable architecture. The measurement of green finance is accomplished in this study through the utilization of a Green Finance Index [46]. Utilizing the entropy approach, the Green Finance Index is computed considering factors like green credit, investments, insurance, bonds, support, funds, and equity.

Environmental Regulation(ER): Environmental regulation represents a pivotal social control mechanism enforced by the authorities in order to safeguard the environment and attain sustainable economic growth with an emphasis on ecological sustainability. It involves mandatory pollution control measures for polluting companies. Essentially, it forces companies to change their existing production patterns by increasing their costs, in the end, creating an outcome advantageous to both the environment and the economy. This research utilizes input and output metrics to gauge the strength of environmental regulation and applies the entropy technique to calculate indicator weights to build an all-encompassing environmental regulation index [47].

#### 5.2.4 Control variables

To further alleviate potential issues such as omitted variable bias and endogeneity, this study, drawing inspiration from existing research, introduces additional factors that could impact the regional energy structure. These factors regarding the extent of local economic progress, the proportion of Secondary industry, population density, patent applications, and the extent of government interference. The specific definitions and calculation methods for each variable are provided in Table 1, while Table 2 presents the descriptive statistical data for the main variables.

**Table 1:** Definitions and computation methods of variables.

Variable representation	Meaning of variables	Method of calculation
post✕treat	Carbon neutrality	post × treat, post is the year dummy variable, and treat is the pilot city dummy variable
mean_ max	The highest carbon pricing value	The annual highest value of carbon trading price
mean_ min	Lowest carbon pricing value	Annual minimum value of carbon trading price
TRE	energy consumption structure	Geometric Mean of Oil and Gas Substitution Coal Index and Non-Fossil Energy Substitution Fossil Energy Index
OGE	Oil and gas substitution coal index	Using the conversion coefficient of standard coal to convert the units of energy consumption into standard coal, the ratio of the sum of the fraction of oil and natural gas consumption to the fraction of coal consumption is calculated
REE	Non-fossil energy substitution index	The proportion of non-fossil energy consumption and the proportion of fossil energy consumption
GFI	Green Finance Index	A comprehensive indicator system was obtained by using the entropy method to calculate green credit, green investment, green insurance, green bonds, green support, green funds, and green equity
ER	Environmental Regulation Index	Choose three distinct metrics: the rate at which sulfur dioxide is eliminated, the rate at which smoke (powder) is removed from dust, and the overall rate at which solid waste is used from each province to develop an all-encompassing measurement framework for the strength of official environmental rules
SEC	Proportion of Secondary industry	The proportion between the Secondary industry and the overall economic production
GDP	Level of economic development	Per capita GDP
POPU	population density	Logarithmic population density
PAT	Number of patent applications	Count of patent filings in the area
GOV	Degree of government intervention	Local government fiscal expenditure/regional GDP

**Table 2:** Descriptive statistics of main variables.

Variable	Obs	Mean	Std. Dev.	Min	Max
TRE	4498	0.158	0.204	0.003	2.343
OGE	4498	0.219	0.474	0	6.162
REE	4498	0.143	0.096	0.04	0.891
GFI	4844	0.321	0.108	0.058	0.64
ER	3462	0.248	1.224	0	35.101
SEC	4498	46.123	11.653	10.7	91
GDP	4498	16.813	1.333	13.335	19.76
POPU	4498	5.935	0.805	1.569	7.882
PAT	4498	7.757	2.132	1.609	12.217
GOV	4498	0.189	0.094	0.043	1.485

Economic Development Level (GDP): This study assesses the economic development of various regions by examining the per capita GDP in yuan, which is obtained from the "China Statistical Yearbook." To simplify the calculations, the per capita regional GDP is computed using the natural logarithm, denoted as GDP.

Population density (population): The main measure of population concentration, derived from the "China Statistical Yearbook," is population density. In a similar manner to GDP, the natural logarithm of population density is calculated for ease of use and referred to as population. Population density is determined by dividing a prefecture-level city's population by its administrative region, illustrating the variance in the extent of human activities within the city.

Proportion of the Secondary Industry (sec): This variable symbolizes the fraction of the Secondary industry to the total economy, sourced from the "China Statistical Yearbook."

Patent applications (patent): Count of patent filings in the area.

Degree of Government Intervention (gov): The main measure of this phenomenon is typically quantified as the ratio of fiscal expenditure by local governments to the regional Gross Domestic Product (GDP).

After performing the interpolation of crucial continuous variables, the descriptive statistics are presented for each variable in Table 2. These statistics include the sample size, mean, standard deviation, as well as the minimum, median, and maximum values.

## 6 Empirical results analysis

### 6.1 Stationarity trend test

**Fig. 1:**
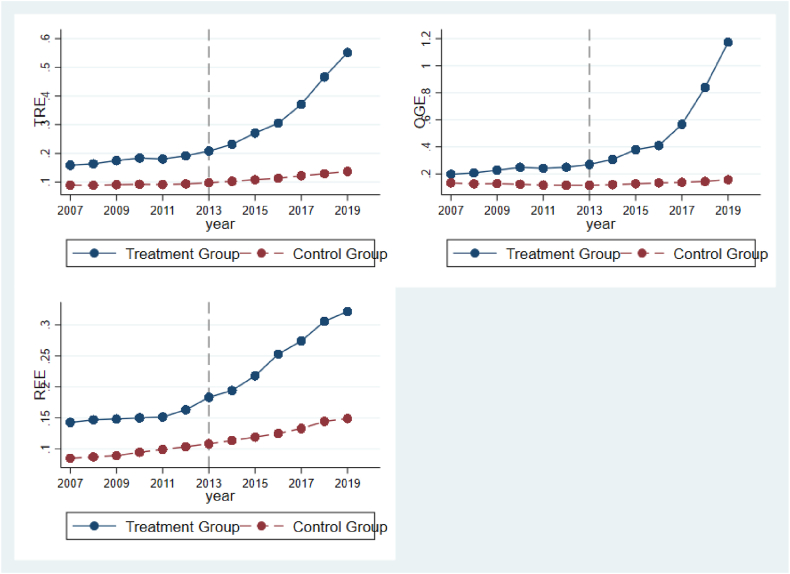
Stationary trend test.

The present research examines the utilization of the start time and urban areas of the emission trading market as virtual factors in formulating the carbon neutrality policy. By conducting a quasi-natural experiment using the differences before and after the carbon emission trading market pilot time in 2013 and the six pilot provinces and cities, we aim to investigate the impact of this policy on optimizing the energy structure. The initiation time of carbon emission rights and the provinces and cities with pilot programs are considered the treatment group, while the time and regions without pilot programs serve as the control group. For the foundational regression analysis, a dual-difference model has been developed. In the baseline analysis, we identify the influence of the carbon emission trading pilot on the energy consumption structure in China. However, it is important to determine whether the variances in the regional energy consumption structure's double substitution process, before and after the carbon emission trading pilot, are solely due to the pilot policy or if there are other unobservable factors at play. Therefore, to validate the outcomes, it is necessary to conduct a parallel trend analysis using a double difference model. The outcomes of this study's double-difference model are depicted in Fig. 1, which shows the results of the pre and post-event trend study for the six periods before and after the event. The analysis reveals that before the pilot policy, there was no significant disparity in the substitution process of energy consumption between the pilot and nonpilot areas. However, following the launch of the carbon emission trading market, the policy's influential impact gradually became evident. From the seventh year, the double substitution process of the regional energy consumption structure significantly accelerated and reached a high level. At the same time, the policy impact of the carbon emission trading trial experienced a notable delay, which can be attributed to institutional inflexibility, habitual behaviors, and the expenses associated with transition. It takes some time for the decision-making of various stakeholders after receiving the policy signal. According to the data, both the treatment and control groups successfully passed the trend test before the implementation of the pilot program. The results of this study demonstrate that the treatment group and the control group exhibited the same development trend before 2013, indicating that the results passed the parallel trend test.

### 6.2 Baseline regression results

This section undertakes an evaluation of the impact of the carbon emissions trading policy on energy structure optimization through the construction of a Difference-in-Differences (DID) model. The estimation results obtained are presented in Table 3. Columns 1, 3, and 5 provide the basic findings, which highlight the effects of the carbon trading pilot on the Oil and Gas Replacement Index (OGE), the Non-Fossil Energy Replacement Index (REE), and the Energy Structure Double Replacement Index (TRE), respectively. Inclusion of control variables is done in columns 2, 4, and 6 in addition to the basic regression. The results indicate a significant positive effect of the pilot carbon emissions trading on the energy usage framework. Specifically, the findings in columns 1, 3, and 5 suggest that the carbon emissions trading market has a positive and significant impact on the energy structure double replacement index, oil and gas substitution index, and non-oil and gas substitution index following the pilot in the designated areas and cities. The regression coefficient for TRE on the carbon neutralization policy variable carbon neutralization (post × treat) is 0.1430, which holds significance at the 1 % threshold. Even with the incorporation of variables such as the proportion of the secondary industry, economic growth stage, population size, density, number of patent filings, and level of governmental involvement in columns 2, 4, and 6, the TRE regression coefficient for the policy factor carbon neutralization (post × treat) remains 0.1370, maintaining its significance at the 1 % threshold. The outcomes clearly demonstrate that, regardless of the inclusion of control variables, the carbon emissions trading market pilot has a remarkable positive influence on the energy consumption structure. In other words, since the implementation of the carbon emissions trading market trial in 2013, there has been a significant improvement in the energy usage framework across the pilot provinces and cities, including Beijing, Shanghai, Tianjin, Chongqing, Hubei, and Guangdong. The carbon emissions trading pilot program is vital for achieving both the total and intensity of carbon dioxide emissions, which are necessary for the attainment of carbon peak and carbon neutrality goals.

**Table 3:** Baseline regression results.

	(1)	(2)	(3)	(4)	(5)	(6)
	TRE	TRE	OGE	OGE	REE	REE
post✕treat	0.143***	0.137***	0.325***	0.314***	0.0652***	0.0600***
	(16.30)	(15.18)	(13.65)	(12.78)	(20.32)	(18.30)
SEC		0.00263***		0.00669***		0.000761***
		(3.98)		(3.74)		(3.19)
GDP		0.0430**		0.0739		0.0453***
		(1.98)		(1.25)		(5.74)
POPU		0.00662		0.0189		0.000979
		(1.31)		(1.38)		(0.53)
PAT		−0.0333***		−0.0806***		−0.0104***
		(-4.79)		(-4.27)		(-4.12)
GOV		−0.108*		−0.308*		−0.0450**
		(-1.81)		(-1.90)		(-2.07)
_cons	0.112***	−0.513	0.155***	−0.900	0.104***	−0.593***
	(15.09)	(-1.53)	(7.67)	(-0.99)	(38.39)	(-4.89)
N	4316	4316	4316	4316	4316	4316
Year FE	YES	YES	YES	YES	YES	YES
City FE	YES	YES	YES	YES	YES	YES
R2	0.178	0.190	0.113	0.123	0.382	0.400
adj. R2	0.107	0.119	0.036	0.046	0.329	0.347
F	66.28	51.67	38.86	30.84	189.2	146.7
p	1.77e-158	3.75e-166	1.63e-93	4.99e-99	0	0

Note: ***, **, and * represent the 1 %, 5 %,and 10 % significance levels respectively. The values in parentheses are t statistics.

### 6.3 Placebo test

**Fig. 2:**
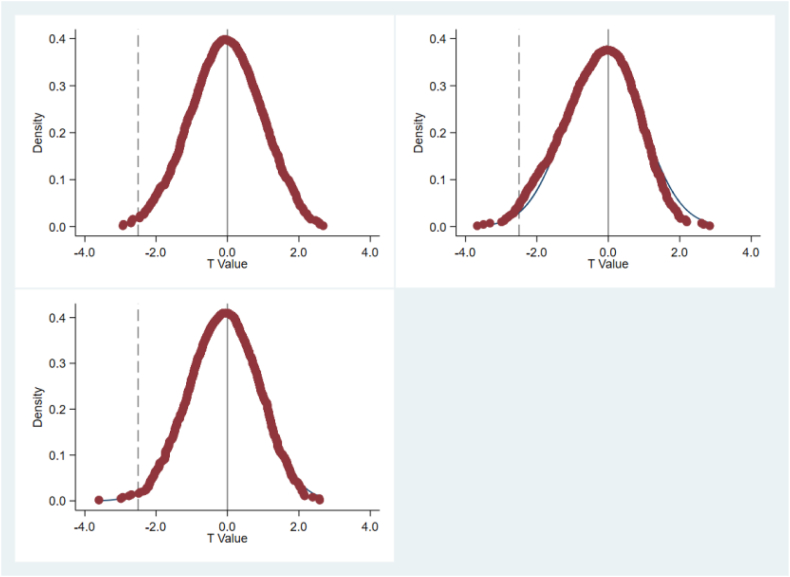
Placebo test (estimated coefficient kernel density estimate).

**Fig. 3:**
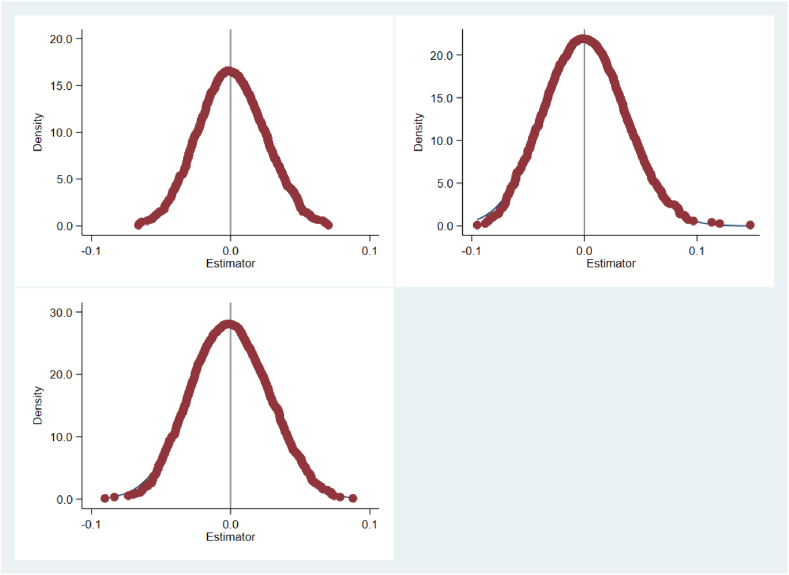
Placebo test (estimated coefficient t-value).

Investigating how much the outcomes are affected by excluded variables and random elements, following the approach of Li and Wang (2016), this study conducts a random "screening" of "carbon-neutral" carbon trading pilot cities and randomly generates pilot times, constructing a two-level random experiment at the time and city levels. Subsequently, based on the baseline regression with the dependent variable as TRE, a regression is carried out according to the random, experiment and to evaluate the dependability of the findings, the likelihood of the predicted coefficients in the initial regression analysis is calculated. To augment the placebo test's effectiveness, the procedure is replicated a thousand times, and the spread of the predicted coefficients for the post × treatment is graphically represented. This is done to verify whether the regional energy consumption structure is remarkably influenced by factors other than "carbon neutrality". If the distribution of the calculated coefficients of post × treat is centered around 0 under random processing, it implies that the model setup does not omit sufficiently important influencing factors. In other words, the effects in the initial baseline analysis are, indeed, the results of the policy focus. Fig. 2, Fig. 3 report the distribution plots of the estimated coefficients, showing that the kernel density estimate and t-values of the calculated values of the false coefficients double difference terms are distributed around 0. The estimated results do not exhibit obvious outliers in the placebo test's estimated coefficients compared to the actual estimated coefficients. Therefore, the estimated results are not significantly biased due to omitted variables, this suggests the absence of a significant issue with omitted variables in the model configuration, maintaining the robustness of the fundamental findings.

### 6.4 Robustness test

#### 6.4.1 Replace the explanatory variable

Considering the existence of carbon emission trading pilot cities and the availability of trading prices, this study further obtained carbon trading prices from the Wind database and calculated the annual average highest and lowest values as two additional indicators to measure the carbon trading pilot policy. The primary regression underwent robustness assessments, yielding results that aligned with the initial regression analysis.

Table 4 presents the forecasted outcomes regarding the influence of the annual average highest carbon price on the coefficients of the energy structure. The baseline results, displayed in the first, third, and fifth columns, depict the effects of the annual average highest carbon price on the oil and gas substitution index (OGE), the non-fossil energy substitution index (REE), and the energy structure double substitution index (TRE), respectively. It is noteworthy that the regression coefficient of the TRE energy structure double substitution index on the policy variable, the annual average highest carbon price, is 0.0139, which is highly significant at the 1 % threshold. In simpler terms, the yearly mean maximum carbon cost has a significant positive impact on energy consumption. When introducing control variables in the second, fourth, and sixth columns, the regression coefficient of TRE on the policy variable, the annual average highest carbon price, remains significant at the 1 % level, with a value of 0.0140. These regression results demonstrate that even when considering the explanatory variable, the annual average highest carbon price still has a substantial beneficial effect on optimizing the structure of energy consumption. This perspective provides additional insights for evaluating the carbon emission trading pilot policy.

**Table 4:** Regression results between the highest annual carbon price and the energy consumption structure coefficient.

	(1)	(2)	(3)	(4)	(5)	(6)
	TRE	TRE	OGE	OGE	REE	REE
mean_ max	0.0139***	0.0140***	0.0398***	0.0398***	0.00403***	0.00401***
	(14.17)	(14.73)	(13.86)	(14.06)	(13.52)	(14.73)
SEC		0.0304***		0.0786***		0.0107***
		(5.08)		(4.41)		(6.22)
GDP		0.303*		0.638		0.164***
		(1.79)		(1.26)		(3.36)
POPU		0.186***		0.483***		0.0683***
		(8.15)		(7.10)		(10.44)
PAT		−0.0471		−0.0154		−0.0457***
		(-1.01)		(-0.11)		(-3.41)
GOV		1.329**		1.500		0.804***
		(2.04)		(0.77)		(4.30)
_cons	−0.404***	−8.258***	−1.460***	−19.82**	0.00195	−3.587***
	(-8.38)	(-2.75)	(-10.35)	(-2.22)	(0.13)	(-4.17)
*N*	703	703	703	703	703	703
Year FE	YES	YES	YES	YES	YES	YES
City FE	YES	YES	YES	YES	YES	YES
*R*^2^	0.422	0.518	0.388	0.470	0.507	0.633
adj. *R*^2^	0.308	0.418	0.267	0.360	0.409	0.557
F	61.12	52.13	53.12	43.00	86.09	83.59
p	9.15e-66	4.39e-84	1.28e-58	2.76e-72	8.41e-86	5.34e-118

Note: ***, **,and * represent the 1 %, 5 %,and 10 % significance levels respectively. The values in parentheses are t statistics.

The outcomes from the estimated analysis on the impact of the lowest annual average carbon price on the coefficients of the energy structure are presented in Table 5. The baseline results, shown in Columns 1, 3, and 5, demonstrate the effects of the lowest annual average carbon price on various substitution indices, namely the oil and gas substitution index (OGE), the non-fossil energy substitution index (REE), and the energy structure double substitution index (TRE), respectively. The regression coefficient of the TRE index, representing the double substitution of energy structure, on the policy variable, the annual average lowest carbon price, is found to be 0.0144, which is highly significant at the 1 % level. In other words, the lowest annual average carbon cost has a considerable positive effect on the energy utilization framework. When control variables are introduced in Columns 2, 4, and 6, the regression coefficient of TRE on the policy variable remains the same, with a significant level of 1 %. These regression results indicate that even after controlling for other variables, the lowest annual average carbon price, which can be used to evaluate the carbon emission trading pilot policy, still has a significant beneficial impact on optimizing the energy consumption framework.

**Table 5:** Regression results between the lowest annual carbon price and the energy consumption structure coefficient.

	(1)	(2)	(3)	(4)	(5)	(6)
	TRE	TRE	OGE	OGE	REE	REE
mean_ min	0.0144***	0.0144***	0.0412***	0.0411***	0.00417***	0.00415***
	(14.70)	(15.29)	(14.37)	(14.60)	(14.05)	(15.32)
SEC		0.0303***		0.0783***		0.0106***
		(5.12)		(4.44)		(6.27)
GDP		0.307*		0.648		0.165***
		(1.83)		(1.30)		(3.43)
POPU		0.185***		0.481***		0.0681***
		(8.20)		(7.14)		(10.53)
PAT		−0.0400		0.00490		−0.0437***
		(-0.86)		(0.04)		(-3.29)
GOV		1.384**		1.656		0.820***
		(2.14)		(0.86)		(4.43)
_cons	−0.421***	−8.414***	−1.506***	−20.25**	−0.00305	−3.629***
	(-8.80)	(-2.84)	(-10.77)	(-2.29)	(-0.21)	(-4.27)
*N*	703	703	703	703	703	703
Year FE	YES	YES	YES	YES	YES	YES
City FE	YES	YES	YES	YES	YES	YES
*R*^2^	0.433	0.529	0.399	0.481	0.516	0.641
adj. *R*^2^	0.321	0.430	0.281	0.373	0.420	0.566
F	63.97	54.28	55.69	44.82	89.32	86.52
p	3.29e-68	1.04e-86	5.84e-61	1.00e-74	3.53e-88	9.61e-121

Note: ***, **,and * represent the 1 %, 5 %,and 10 % significance levels respectively. The values in parentheses are t statistics.

#### 6.4.2 Controlling for province-year fixed effects

To address concerns about potential omitted variables in the regression conclusions, this research considers both provincial and annual fixed factors to lessen the impact of constant, unchangeable elements at the provincial scale over time, as well as the effect of temporal trends on the precision of regression outcomes. This fixed effect can control for factors where province characteristics and province resource endowments do not change over time, thus reducing bias in the conclusions. The results of the research have not been altered. The results of the regression analysis are displayed in Table 6.

**Table 6:** Controlling for province-year fixed effects.

	(1)	(2)	(3)	(4)	(5)	(6)
	TRE	TRE	TRE	TRE	TRE	TRE
post✕treat	0.144***	0.141***	0.142***	0.0388***	0.0440***	0.0528***
	(16.42)	(15.79)	(15.68)	(4.00)	(4.67)	(6.31)
SEC	0.00277***	0.00222***	0.00221***	0.00442***	−0.00107**	−0.00301***
	(5.10)	(3.39)	(3.36)	(7.05)	(-2.51)	(-9.18)
GDP		0.0294	0.0311	−0.000283	0.0412***	−0.00946**
		(1.50)	(1.55)	(-0.01)	(6.47)	(-2.50)
POPU			0.00196	0.0182***	0.0150***	0.0125***
			(0.39)	(3.82)	(3.03)	(3.08)
PAT				0.00000362***	0.00000280***	0.00000328***
				(22.16)	(17.77)	(23.20)
GOV					−0.164***	−0.147***
					(-3.08)	(-3.89)
_cons	−0.0217	−0.469	−0.506	−0.221	−0.599***	0.347***
	(-0.79)	(-1.57)	(-1.61)	(-0.75)	(-5.28)	(5.42)
N	4316	4316	4316	4316	4316	4316
Year FE	YES	YES	YES	YES	NO	NO
City FE	YES	YES	YES	YES	YES	NO
R2	0.184	0.184	0.184	0.274	0.202	
adj. R2	0.112	0.113	0.113	0.210	0.134	
F	63.85	59.76	56.03	88.14	168.1	
p	2.94e-163	7.86e-163	5.74e-162	2.46e-260	5.11e-191	0

Note: ***, **, and * represent the 1 %, 5 %,and 10 % significance levels respectively. The values in parentheses are t statistics.

#### 6.4.3 Removal of outliers

The annual average lowest carbon price, which can be used to evaluate the carbon emission trading pilot policy, still has a considerable beneficial effect on optimizing the energy consumption structure. To enhance the credibility of empirical results and prevent outliers from interfering with regression outcomes, the study removes extreme values within the 2.5 percentiles. The regression results after excluding outliers show no significant differences in coefficient values, signs,or significance levels compared to the fundamental regression model. This confirms that the carbon emission trading pilot policy encourages energy structure optimization, and the coefficients and levels of significance align with the initial regression findings, thereby confirming the dependability of the estimated outcomes. The results of the regression analysis are displayed in Table 7.

**Table 7:** Removal of outliers.

	(1)	(2)	(3)	(4)	(5)	(6)
	TRE	TRE	OGE	OGE	REE	REE
post✕treat	0.0348***	0.0263***	0.0602***	0.0525***	0.0201***	0.0193***
	(24.66)	(16.01)	(24.05)	(18.84)	(19.80)	(16.82)
SEC		0.0000159		−0.000127		−0.0000518
		(0.14)		(-0.64)		(-0.62)
GDP		−0.0166***		−0.0771***		0.0132***
		(-4.02)		(-10.99)		(4.53)
POPU		−0.00521***		−0.0132***		−0.00653***
		(-3.70)		(-5.55)		(-6.55)
PAT		−0.000486		0.000989		0.00163*
		(-0.35)		(0.42)		(1.65)
GOV		−0.106***		−0.156***		−0.106***
		(-5.24)		(-4.56)		(-7.22)
_cons	0.109***	0.415***	0.139***	1.459***	0.103***	−0.0690
	(95.84)	(6.38)	(69.19)	(13.21)	(128.25)	(-1.51)
N	3895	2802	3897	2820	3890	2731
Year FE	YES	YES	YES	YES	YES	YES
City FE	YES	YES	YES	YES	YES	YES
R2	0.576	0.619	0.325	0.426	0.734	0.782
adj. R2	0.534	0.572	0.259	0.355	0.709	0.756
F	370.3	225.0	131.4	103.5	756.1	485.3
p	0	0	6.08e-291	5.34e-286	0	0

Note: ***, **, and * represent the 1 %, 5 %,and 10 % significance levels respectively. The values in parentheses are t statistics.

#### 6.4.4 Reduction of control variables

This study employs a method to add and remove control variables for further examination. The findings indicate that the inclusion or exclusion of control variables in the initial regression analysis does not alter the favorable influence of carbon neutrality policies on the double substitution index of the energy structure. Factors such as regional economic development, the proportion of the Secondary industry, population density, patent applications, Government intervention, and other control variables exhibit no significant impact on this positive relationship. This outcome provides additional validation for the credibility of the baseline regression findings, which are presented in Table 6.

### 6.5 Further mechanism analysis

In order to further support the hypothesis presented in Hypothesis 2 (H2) that the pilot program for carbon emissions trading promotes the transformation of regional energy consumption structures through the improvement of green finance, this study utilizes the double difference method to interact the baseline explanatory variables with green finance. The results of the regression analysis can be found in Table 8. Columns 1, 3, and 5 indicate that the introduction of the carbon emissions trading market in the pilot provinces and cities has led to a significant enhancement of the energy structure, as evidenced by the double substitution index, oil and gas substitution index, and nonoil and gas substitution index. The coefficient for the regression of the energy structure double substitution index (TRE) on the policy variable green carbon neutrality (carbon neutrality GFI) is 0.3150, which is statistically significant at the 1 % level. When control variables such as the proportion of the secondary industry, the level of economic development, the population density, patent applications, and Government intervention are included in columns 2, 4, and 6, the coefficient of the regression of TRE on the policy variable carbon neutrality (carbon neutrality GFI) is 0.2970, which is also statistically significant at the 1 % level. The implementation of the pilot policy for carbon emissions trading appears to have a significant impact on the energy usage framework, provided that green finance is properly implemented. Following the initiation of the carbon emissions trading market trial in 2013, the introduction of green finance in the pilot provinces and cities of Beijing, Shanghai, Tianjin, Chongqing, Hubei, and Guangdong has resulted in a noticeable improvement in the energy consumption structure.

**Table 8:** Regression results of the interaction between carbon neutrality and green finance.

	(1)	(2)	(3)	(4)	(5)	(6)
	TRE	TRE	OGE	OGE	REE	REE
post✕treat	0.0178	0.0205	−0.0112	−0.00619	0.0196*	0.0187*
	(0.59)	(0.67)	(-0.14)	(-0.07)	(1.78)	(1.69)
GFI	−0.0511	−0.0374	−0.0352	0.00196	−0.0621	−0.0522
	(-0.49)	(-0.36)	(-0.13)	(0.01)	(-1.64)	(-1.39)
GFI(post✕treat)	0.315***	0.297***	0.839***	0.809***	0.116***	0.106***
	(4.28)	(3.95)	(4.21)	(3.97)	(4.32)	(3.89)
SEC		0.00246***		0.00624***		0.000700***
		(3.73)		(3.49)		(2.93)
GDP		0.0472**		0.0856		0.0467***
		(2.17)		(1.45)		(5.92)
POPU		0.0113**		0.0323**		0.00240
		(2.18)		(2.30)		(1.28)
PAT		−0.0304***		−0.0719***		−0.00960***
		(-4.34)		(-3.78)		(-3.78)
GOV		−0.107*		−0.306*		−0.0440**
		(-1.79)		(-1.88)		(-2.03)
_cons	0.125***	−0.609*	0.164**	−1.204	0.120***	−0.612***
	(4.51)	(-1.81)	(2.17)	(-1.32)	(11.84)	(-5.02)
*N*	4316	4316	4316	4316	4316	4316
Year FE	YES	YES	YES	YES	YES	YES
City FE	YES	YES	YES	YES	YES	YES
*R*^2^	0.182	0.193	0.117	0.127	0.385	0.402
adj. *R*^2^	0.111	0.122	0.040	0.049	0.332	0.349
F	59.01	47.52	35.18	28.76	165.9	133.2
p	7.40e-161	5.41e-168	3.59e-96	2.70e-101	0	0

Note: ***, **,and * represent the 1 %, 5 %,and 10 % significance levels respectively. The values in parentheses are t statistics.

In order to further examine the hypothesis put forth in Hypothesis 3 (H3) that the pilot carbon emissions trading program promotes the transformation of regional energy consumption patterns by encouraging public adoption of low-carbon behavior through environmental regulations, this study utilizes the double-difference method to analyze the interaction between baseline explanatory variables and environmental regulations. The results of the regression analysis are presented in Table 9. The findings in columns 1, 3, and 5 indicate that the launch of the carbon emissions trading market in pilot provinces and cities, driven by environmental laws, significantly increases the adoption of green carbon neutrality, as well as the substitution of oil and gas, and non-oil and gas energy sources. The coefficient for the regression analysis of the energy structure double substitution index (TRE) on the policy variable of environmental carbon neutrality (carbon neutrality environmental regulation) is 0.0565, which is statistically significant at the 1 % level. Additionally, when controlling for other variables such as the proportion of Secondary industry, the level of economic development, the population density, patent applications, and government intervention in columns 2, 4, and 6, the coefficient of regression of TRE on the policy variable of carbon neutrality (carbon neutrality GFI) is 0.0557, also significant at the 1 % level. These results suggest that environmental regulations can be a powerful tool in promoting positive changes in the energy consumption structure towards carbon neutrality. Since the initiation of the carbon emissions trading market trial in 2013, the energy usage framework in the test provinces and cities of Beijing, Shanghai, Tianjin, Chongqing, Hubei, and Guangdong has been influenced and improved by environmental rules that shape societal behavior.

**Table 9:** Regression results of the interaction between carbon neutrality and environmental regulation.

	(1)	(2)	(3)	(4)	(5)	(6)
	TRE	TRE	OGE	OGE	REE	REE
post✕treat	0.0485***	0.0451***	0.0867***	0.0814***	0.0231***	0.0194***
	(11.31)	(10.41)	(7.67)	(7.09)	(10.88)	(9.25)
ER	−0.000150	0.000139	−0.000165	0.000122	−0.0000180	0.000291
	(-0.21)	(0.19)	(-0.09)	(0.06)	(-0.05)	(0.85)
ER(post✕treat)	0.0565***	0.0557***	0.140***	0.141***	0.0163***	0.0153***
	(7.67)	(7.62)	(7.23)	(7.26)	(4.45)	(4.34)
SEC		0.000342		0.00203***		−0.0000849
		(1.38)		(3.10)		(-0.71)
GDP		0.00936		−0.0552**		0.0267***
		(1.15)		(-2.55)		(6.78)
POPU		−0.00808***		−0.0180***		−0.00772***
		(-4.03)		(-3.38)		(-7.98)
PAT		−0.00425*		−0.0107*		0.00153
		(-1.76)		(-1.67)		(1.31)
GOV		−0.0637***		−0.130**		−0.0275***
		(-3.15)		(-2.43)		(-2.81)
_cons	0.102***	0.0177	0.139***	1.083***	0.0987***	−0.276***
	(36.91)	(0.15)	(19.18)	(3.36)	(72.34)	(-4.69)
*N*	3297	3297	3297	3297	3297	3297
Year FE	YES	YES	YES	YES	YES	YES
City FE	YES	YES	YES	YES	YES	YES
*R*^2^	0.251	0.264	0.080	0.090	0.549	0.580
adj. *R*^2^	0.182	0.194	−0.005	0.004	0.507	0.540
F	67.36	53.92	17.47	14.83	244.7	207.7
p	1.82e-176	4.70e-183	5.18e-45	1.73e-48	0	0

Note: ***, **,and * represent the 1 %, 5 %,and 10 % significance levels respectively. The values in parentheses are t statistics.

## 7 Conclusion and policy recommendations

This scholarly study utilizes panel data from various provinces in China spanning from 2007 to 2020 in order to examine the effects of carbon emission trading on energy system optimization. The study draws three key conclusions: Firstly, the implementation of the pilot carbon emission trading scheme has significantly expedited the process of regional energy consumption structure transformation. This is evident through the dual substitution of "oil and gas replacing coal" and "non-fossil energy replacing fossil energy." The pilot policy has effectively reduced carbon emissions and carbon emission intensity in the pilot regions, showcasing a noteworthy reduction in carbon emissions. Secondly, the development of green finance within the carbon emission trading pilot provinces holds significant influence over the level of carbon emissions in these regions. By promoting regional green finance policies, the performance of low-carbon economic transformation can be enhanced to a certain extent. The development of green finance can yield positive low-carbon effects, improve energy efficiency, promote environmental harmony, and contribute to the optimization of regional energy consumption structure. Lastly, the implementation of carbon emission trading pilots stimulates positive and substantial impacts on the optimization of energy consumption structure through environmental regulations. The establishment of these pilots prompts increased public awareness and engagement in ecological environment supervision, as well as encourages low-carbon consumption, green travel, and energy conservation within the pilot regions. Consequently, this further drives the transformation of energy consumption structure. With the implementation of environmental regulations within the region, public participation in environmental protection can be increased to a certain extent, thereby promoting the low-carbonization of the pilot regions, improving energy efficiency, fostering environmental harmony, and playing a vital role in optimizing regional energy consumption structure.

From a macro perspective, the role of carbon neutrality in optimizing the energy structure has not been fully leveraged, and the current green finance system under the dual carbon target remains imperfect. Therefore, it is necessary to further promote the energy revolution, strengthen the efficient and clean utilization of coal, and expedite the planning and construction of a new energy system to achieve the dual carbon development goals. Drawing upon the aforementioned research findings, this scholarly article posits three policy recommendations: Firstly, it is necessary to continue improving the carbon emission trading market to promote the optimization of the energy structure. Recognizing the beneficial impact of carbon emission trading on energy consumption optimization, it is crucial to accelerate the establishment of carbon emission trading markets in pilot areas. The development of carbon trading regulations should take into account the carbon emission scale and economic development level of pilot areas, and be targeted in the promotion of carbon trading markets. Pilot policies should prioritize cities with lower economic levels and strengthen inter-regional coordination and cooperation. It is important to strengthen the construction of a unified national carbon trading market system to ensure the synchronized operation of carbon trading pilots with the national carbon market, and accelerate the integration of carbon trading pilots into the national market. Secondly, we need to optimize the energy consumption structure through green finance. This involves intensifying the development of green finance, enhancing the level of green technology innovation, and promoting the transformation of traditionally inefficient and outdated fossil fuel production methods such as coal towards cleaner and more efficient production methods. It is necessary to fully utilize financial resource allocation, innovate green finance development models, and guide capital towards technological innovation projects that facilitate the green and low-carbon adjustment of the energy consumption structure. This will help correct the current imbalance in energy consumption and promote the development and application of new energy sources. Thirdly, we need to improve the environmental regulatory system to increase energy efficiency. For regions with relatively low levels of environmental regulation, it is necessary to appropriately increase the intensity of regulation to stimulate green transformation and maintain a sustained reduction in healthy energy intensity. Given the current low level of environmental pollution control, the government should appropriately strengthen environmental regulation, raise pollution discharge fees, and increase environmental law enforcement efforts. However, overly strict command-and-control environmental policies may not be conducive to reducing healthy energy intensity. Therefore, it is necessary to adjust the level of environmental regulation within a threshold range to better stimulate its role in optimizing the energy consumption structure.

Due to the current provincial nature of China's carbon emission trading policy, obtaining policy data at the city and industry levels is challenging. As a result, this article is unable to conduct more specific analysis. However, as China's carbon trading pilot continues to develop and improve, testing the impact of carbon trading on energy structure at the city and industry levels would allow for a more thorough examination of the regional variations in carbon trading policies. This would provide better empirical evidence for the establishment of carbon markets and the optimization of regional energy structures. Furthermore, to address potential issues with sample selection and omitted variables in the DID method, future research could employ quantitative simulation and balanced panel methods to enhance the accuracy and reliability of the findings.

Data Statement for this article: This is an open access article, and the authors are willing to share the dataset and estimate code in Excel format with anyone who wishes to replicate the results of this study. The explained variables in this paper refer to the practice of [44] and compile three alternative indices of energy structure to measure the degree of optimization of energy structure. The explanatory variable carbon neutrality in this paper is obtained by the author manually compiling the list of provinces participating in the pilot carbon emission trading scheme from the government-recognized website, and then constructing the virtual variable combined with the implementation time of the pilot policy. As for the explanatory variable in the robustness test, the carbon trading price is obtained by retrieving the annual carbon trading price from Wind database. The data of regulatory variables and control variables in this paper mainly come from CSMAR database, WIND database, EPS platform, China Energy Statistical Yearbook, China Environmental Statistical Yearbook and various provincial statistical yearbooks. However, it should be noted that due to the availability of data, the data of Chinese provinces in this paper are not included in the data of China's Tibet, Hong Kong, Macao and Taiwan.

## Funding

This paper is supported by 10.13039/501100001809National Natural Science Foundation of China (72341014).

## CRediT authorship contribution statement

**Jia Songbo:** Writing – original draft, Conceptualization. **Zhu Xiaowen:** Writing – original draft, Software. **Gao Xin:** Writing – original draft, Supervision, Formal analysis. **Yang Xiaotong:** Writing – review & editing.

## Declaration of Competing Interest

The authors declare that they have no known competing financial interests or personal relationships that could have appeared to influence the work reported in this paper.
